# Analysis of the Operation Mechanism of Superjunction in RC-IGBT and a Novel Snapback-Free Partial Schottky Collector Superjunction RC-IGBT

**DOI:** 10.3390/mi15010073

**Published:** 2023-12-29

**Authors:** Song Yuan, Yichong Li, Min Hou, Xi Jiang, Xiaowu Gong, Yue Hao

**Affiliations:** 1 The Key Laboratory of Ministry of Education for Wide Bandgap Semiconductor Materials and Devices, School of Microelectronics, Xidian University, Xi’an 710071, China; syuan@xidian.edu.cn (S.Y.); 18127211600@163.com (Y.L.); houmminy@gmail.com (M.H.); xwgong@xidian.edu.cn (X.G.); yhao@xidian.edu.cn (Y.H.); 2 The Guangzhou Institute of Technology, Xidian University, Guangzhou 510555, China

**Keywords:** superjunction, RC-IGBT, snapback, Schottky contact

## Abstract

This paper explores the operation mechanism of the superjunction structure in RC-IGBTs based on carrier distribution and analyzes the advantages and challenges associated with its application in RC-IGBTs for the first time. A Partial Schottky Collector Superjunction Reverse Conduction IGBT (PSC-SJ-RC-IGBT) is proposed to address these issues. The new structure eliminates the snapback phenomenon. Furthermore, by leveraging the unipolar conduction of the Schottky diode and its fast turn-off characteristics, the proposed device significantly reduces the turn-off power consumption and reverse recovery charge. With medium pillar doping concentration, the turn-off loss of the PSC-SJ-RC-IGBT decreases by 54.1% compared to conventional superjunction RC-IGBT, while the reverse recovery charge is reduced by 52.6%.

## 1. Introduction

Power semiconductor devices have been widely used in modern power electronic systems to improve system efficiency as power conversion equipment. The Insulated Gate Bipolar Transistor (IGBT) as a mainstream power device applied in medium high-voltage applications is continuously expanding its application area due to the improvement of the basic structure. The widely used advancements in recent years about IGBTs are based on the following basic structures: (1) trench technology [1], compared with planar IGBTs, trench IGBTs can relieve the current crowding effect when the cell pitch is reduced to achieve lower on-state voltage, and (2) field stop (FS) technology (also known as soft punch through (SPT) or light punch through) [2,3]. After the field stop (FS) IGBT is applied in power electronics, it has almost been the only IGBT structure compared with the punch through IGBT and the nonpunch through IGBT in modern power electronics area, due to much a thinner drift region and an optimized anode doping profile, the FS IGBT is able to achieve very low on-state voltage and switching loss, which are the key parameters related to IGBTs’ power loss. (3) In addition, via the injection enhancement effect (injection-enhanced gate transistor (IEGT), carrier store trench bipolar transistor (CSTBT), etc.), created by adding a carrier-stored layer (CSL) beneath the P-base or optimizing the trench structure, the carrier concentration near the emitter side can be significantly increased, and this carrier distribution profile can reduce the on-state voltage without any adverse effect on the turn-off transient [4,5]. Some of the most advanced IGBTs are now incorporating all these three innovations or introducing backside engineering to further improve the device performance, many researchers have carried out a lot of work in this area to match the high efficiency, low power loss requirement of power devices [6,7,8]. However, the field has reached a virtual limit, with very little improvement in the years before the superjunction structure was proposed. An excellent technology, the superjunction structure can be applied to IGBT devices, and a great deal of research has been conducted in this area. Since the N/P pillar structure provides a quick and complete depletion for the drift region, an ultralow Eoff is obtained. This means that the superjunction can effectively optimize the trade-off between the on-state voltage drop and switching losses in IGBT [9,10,11,12,13,14,15,16].

In practical circuit applications, IGBTs are commonly used in parallel with a fast recovery diode acting as a freewheeling diode. To improve integration, reverse conduction IGBT, also known as RC-IGBT, which integrates the IGBT and the freewheeling diode on the same chip, has been proposed [17,18,19,20]. However, the unique structure of the RC-IGBT can lead to the snapback phenomena [21,22,23]. To alleviate this problem, numerous studies have been conducted worldwide, including the floating-p RC-IGBT [24], dielectric isolation RC-IGBTs [25], dual-gate RC-IGBTs [26], alternating N/P RC-IGBT [27], tunneling RC-IGBTs [28], and so on.

In superjunction IGBTs, due to their unique collector structures, there have been relatively few studies on superjunction RC-IGBTs, and some theoretical studies only discuss the advantages of the superjunction in RC-IGBT; no basic operation mechanism has been revealed [29,30,31]. With the Sentaurus TCAD tool, this paper analyzed the basic working mechanism of a superjunction structure in RC-IGBT based on the carrier distribution during device operation. The way snapback phenomena and power loss performance are influenced by the pillar doping concentration is discussed. Furthermore, issues associated with the application of the superjunction in RC-IGBT are discussed. In order to address these concerns, a novel Partial Schottky Collector Superjunction Reverse Conduction IGBT (PSC-SJ-RC-IGBT) is proposed and studied, and the new structure eliminates the snapback phenomenon and achieves ultra-low power loss and reverse recovery charge by utilizing the unipolar conduction of the Schottky diode and its rapid turn-off characteristics. Other researchers have also proposed new back-side engineering methods to improve the performance of IGBTs, with DCT RC-IGBT eliminating the snapback phenomena with two collector trenches [32]; in addition, trench-isolated SJ-IGBTs improve the SJ-IGBTs with back-side trenches [33] and RB-SJ-IGBTs improve the performance of the bidirectional IGBT [34]. Compared with these structures, the PSC-SJ-RC-IGBTs have three different innovation points: 1. The trench structure with N/P pillars can block the electrons’ flow path at the beginning of the device’s turn-on; this eliminates the snapback phenomena in superjunction RC-IGBTs. The trenches are connected to the collector electrode with no material or doping profile requirements. 2. A P-Schottky contact is introduced to the back side, and due to the unipolar and bipolar conduction present in the superjunction RC-IGBT at the same time, the disadvantage of the unipolar conduction of Schottky diode on the on-state voltage of IGBTs can be greatly relieved, and the turn-off loss can be significantly reduced; consequently, the Vce-Eoff tradeoff of the new superjunction RC-IGBT can be improved. 3. With the P-Schottky contact, the unipolar conduction characteristics of the Schottky diode is introduced into the device, and the reverse recovery characteristics can be improved.

## 2. Operation Mechanism of Superjunction in RC-IGBTs and the New PSC-SJ-RC-IGBTs

**Figure 1:**
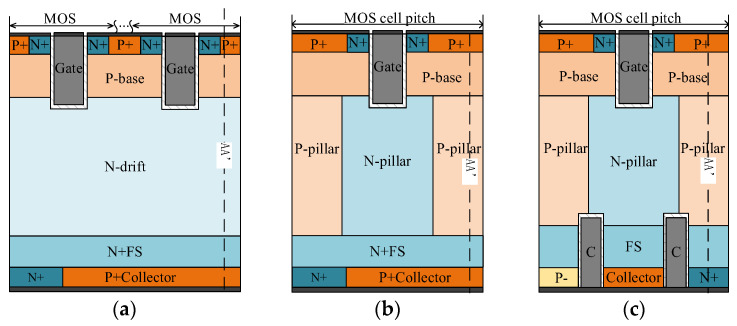
Schematic cross-sections of the (**a**) Con-RC-IGBT, (**b**) Con-SJ-RC-IGBT and (**c**) PSC-SJ-RC-IGBT.

Figure 1 illustrates the device structures of conventional RC-IGBTs (Con-RC-IGBTs), conventional superjunction RC-IGBTs (Con-SJ-RC-IGBTs), and the new Partial Schottky Collector Superjunction Reverse Conduction IGBTs (PSC-SJ-RC-IGBTs) with a voltage rating of 1200 V. Compared to the SJ-RC-IGBT, in the PSC-SJ-RC-IGBT, there are two trenches present on the collector side and the electrodes within the trenches are connected to the collector electrode. Additionally, a portion of the N+ region is replaced with a P-region, enabling the formation of a Schottky diode with the P- region. As a result, the device’s body diode structure resembles a PiN diode in parallel with a Schottky diode, effectively reducing the reverse recovery charge during reverse recovery. Moreover, during turn-off, the drift region carriers can be quickly extracted, further reducing the tail current under inductive loads and minimizing turn-off power consumption. The field stop layer is formed at the bottom of the superjunction as the drift region of the PiN diode during the freewheeling of the PSC-SJ-RC-IGBT. The physical models applied in the simulation include Mobility (DopingDep HighFieldsat Enormal), EffectiveIntrinsicDensity (OldSlotboom), Recombination (SRH (DopingDep) Auger Avalanche (Eparal)) and the Schottky Barrier Tunneling (SBT) model.

Since the drift region of the SJ-RC-IGBTs and PSC-SJ-RC-IGBTs consists of alternately arranged P-pillars and N-pillars, replacing the lightly doped N-region. The thickness of the drift region in the Con-RC-IGBT is 120 μm with a doping concentration of 5 × 10^13^ cm^−3^. The other two IGBTs’ drift region had thicknesses of 100 μm, while the widths of the N-pillars and P-pillars are 2.5 μm. The doping concentration in the superjunction structure varies from 1 × 10^14^ cm^−3^ to 5 × 10^15^ cm^−3^. Other parameters are shown in Table 1.

### 2.1. Breakdown Characteristics

**Figure 2:**
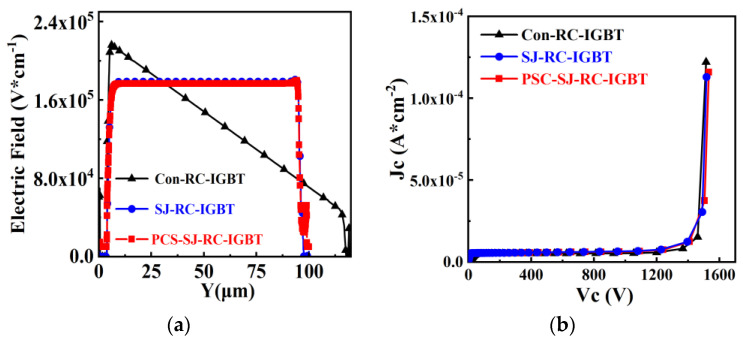
Breakdown characteristics of Con-RC-IGBT, Con-SJ-RC-IGBT and PSC-SJ-RC-IGBT: (**a**) electric field distribution along AA’; (**b**) I-V curves.

Figure 2a illustrates the electric field distribution in the drift region of both Con-RC-IGBT and the two SJ-RC-IGBT devices at the point of breakdown. For Con-RC-IGBT, a conventional trapezoidal electric field distribution can be observed, with electric field strength decreasing gradually from the emitter side to the field stop layer. On the other hand, the two RC-IGBTs with superjunction structures employ a rectangular electric field distribution, with a nearly constant electric field strength throughout the drift region. Thus, under the same drift region length, RC-IGBTs with superjunction structures exhibit a higher breakdown voltage compared to Con-RC-IGBTs. To achieve an equivalent rating, Con-RC-IGBT requires a thicker device structure, as depicted in Figure 2b, with a thickness of 120 μm, whereas SJ-RC-IGBT has a thickness of only 100 μm, yet both show a similar blocking voltage of approximately 1600 V; the leakage current density of the three structures are also at the same level, which means the backside structures have no disadvantages regarding the reverse block characteristics of the IGBTs.

**Figure 3:**
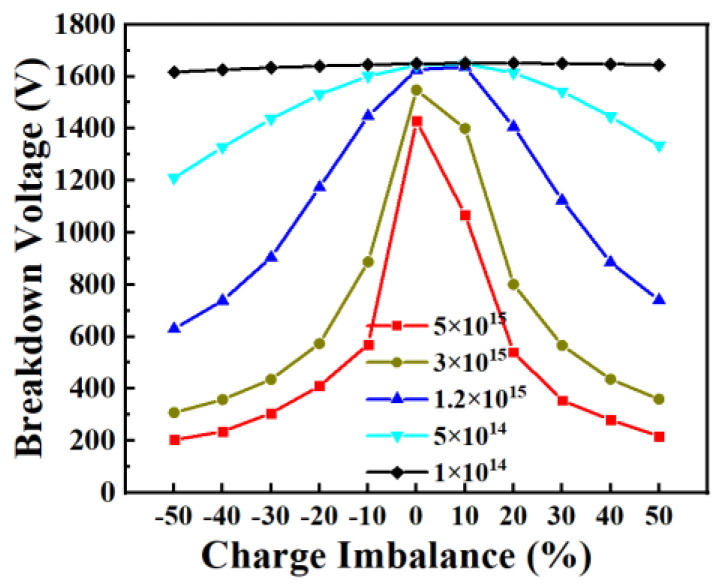
Influence of charge imbalance toward BV.

However, the blocking voltage of the superjunction structure is sensitive to net charge balance conditions. Only when both the N and P pillars are fully depleted can a rectangular electric field distribution be achieved in the drift region to ensure the desired blocking capability. As shown in Figure 3, the higher the doping concentration of the pillars, the more sensitive the device blocking voltage to the charge imbalance. This is because the net imbalance charge in the highly doped pillars is much higher than the charge in lower-doped pillars, and the remained high-density carriers in the high-doped drift region significantly affects the electric file distribution, which leads to a remarkable breakdown voltage decrease [35,36,37].

**Figure 4:**
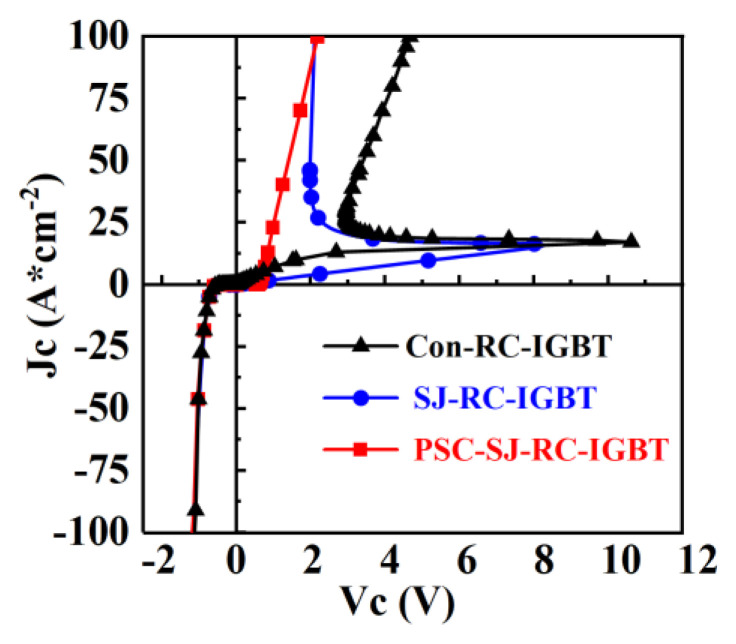
Forward and reverse conduction characteristics of Con-RC-IGBT, Con-SJ-RC-IGBT and PSC-SJ-RC-IGBT.

The snapback phenomenon poses a significant adverse effect on RC-IGBTs, as the sudden voltage snapback with an increasing current leads to negative resistance in IGBTs. However, the negative resistance characteristic is limited the parallel of IGBTs, so the snapback phenomena must be eliminated in RC-IGBTs. As shown in Figure 4, both conventional RC-IGBTs and SJ-RC-IGBTs exhibit snapback. The new PSC-SJ-RC-IGBT eliminates the snapback phenomena.

### 2.2. Forward Conduction Characteristics

#### 2.2.1. Snapback Mechanism

**Figure 5:**
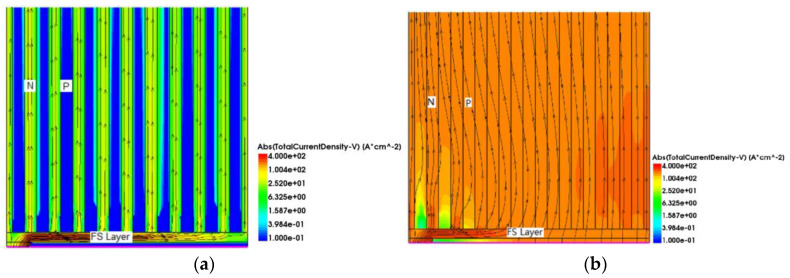
Current distribution of Con-SJ-RC-IGBT during forward conduction at (**a**) J_C_ = 1 A/cm^2^ and (**b**) J_C_ = 100 A/cm^2^.

As shown in Figure 5, because RC-IGBT devices initially operate in unipolar mode, only the MOSFET conducts the current. When the applied voltage on the P+ collector exceeds the built-in potential of the PN junction, the PN junction is positively biased and the IGBT transitions into the bipolar mode. At this point, a large number of carriers are injected into the drift region, resulting in conductivity modulation and rapid reduction in the conduction voltage drop. This unique snapback phenomenon is observed in RC-IGBTs. Particularly for modern field-stop IGBT devices, the doping level of the field-stop layer is relatively high, leading to more severe snapback, as shown in Figure 5b. Furthermore, under a certain current density, the voltage drop across the PN junction near the N-region is not high enough to turn on the junction, which means that no holes are injected into the drift region here, a part of the P-collector region is in the off-state and barely any current is conducted through this region, resulting in the underutilization of the collector area.

**Figure 6:**
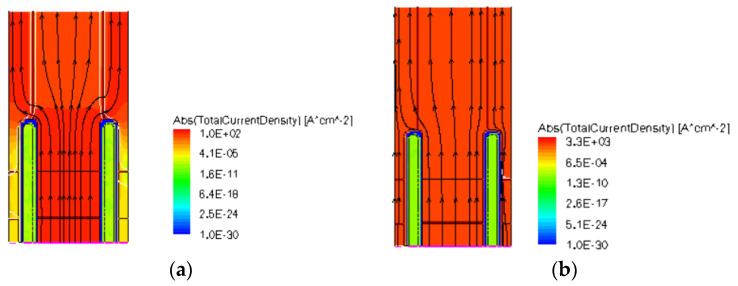
Current distribution of PSC-SJ-RC-IGBT during forward conduction at (**a**) J_C_ = 1 A/cm^2^ and (**b**) J_C_ = 100 A/cm^2^.

For PSC-SJ-RC-IGBT, as shown in Figure 6, during the early stage of forward conduction, the current path between the N-type pillar and the N-collector electrode is separated by the P-type pillar and the trench; consequently, there is no current flowing through the MOSFET section when the device initially conducts. Only the intermediate region of the collector enters the low-level injection state, and the current primarily flows through the IGBT region. As the collector voltage continues to rise, the collector junction transitions into the high-level injection state, with a large number of carriers filling the drift region and entering the sidewalls of the trenches. Both the entire drift region and the sidewalls of the trenches carry current, leading to the full turn-on of the device. Compared to the Con-SJ-RC-IGBT, there is no non-conducting collector region, thereby enhancing the effective utilization of the device area.

#### 2.2.2. Pillar Doping Influence

SJ-RC-IGBTs employ superjunction structures characterized by charge compensation effects. In the case of charge balance, the P and N pillars can achieve high doping concentrations under certain blocking capabilities. When the doping concentration of the P and N pillars is high, the conductivity modulation effect caused by hole injection will be affected, which means the static and switching characteristics will also change.

**Figure 7:**
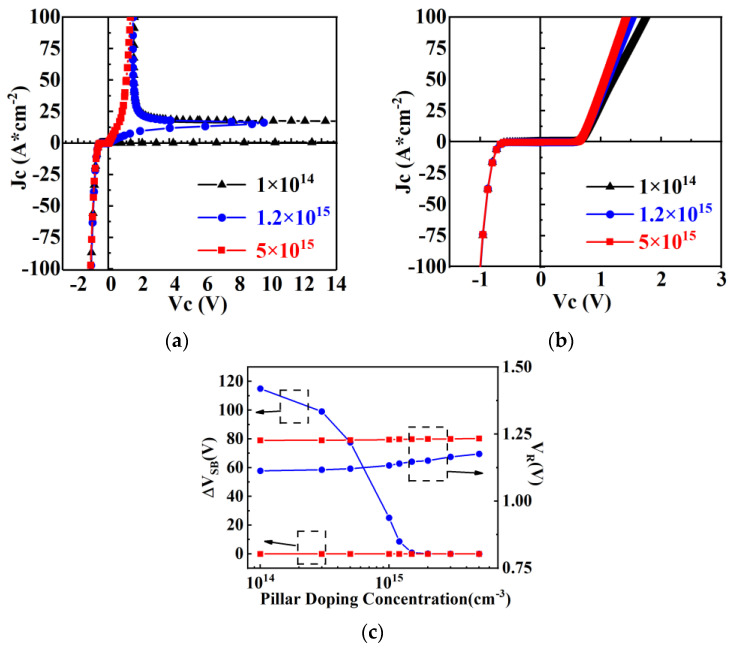
(**a**) Conduction characteristics of Con-SJ-RC-IGBT; (**b**) conduction characteristics of PSC-SJ-RC-IGBT; (**c**) reverse voltage drop (J_C_ = 100 A/cm^2^) and snapback voltage vs. pillar doping concentration (red: PSC-SJ-RC-IGBT; blue: Con-SJ-RC-IGBT).

As shown in Figure 7, the conduction characteristics of the Con-SJ-RC-IGBT and PSC-SJ-RC-IGBT vary with the doping concentration of the P and N pillars. From the graph, no snapback phenomena are observed in PSC-SJ-RC-IGBT. Under the same device parameters, the forward conduction characteristics of the Con-SJ-RC-IGBT are greatly influenced by the doping concentration of the P and N pillars. As the doping concentration of the P and N pillars increases, the snapback phenomena of the Con-SJ-RC-IGBT gradually decreases and eventually disappears. The forward voltage drop of the two devices decreases as the doping concentration increases. On the other hand, the reverse conduction capability of the devices is not significantly affected by the doping concentration of the P and N pillars. This phenomenon is believed to be caused by the influence of doping concentration on the conductivity modulation effect.

**Figure 8:**
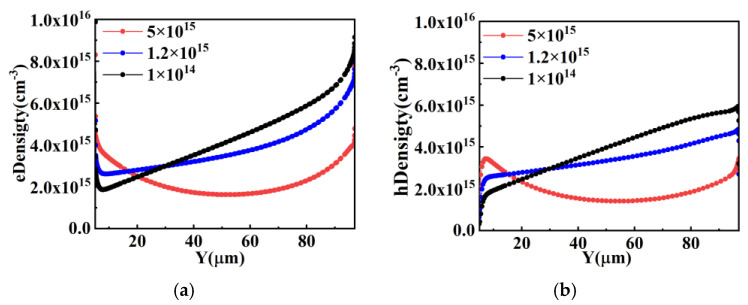
Minority carrier density distribution during forward conduction in the superjunction structure: (**a**) electron density distribution in P-pillar and (**b**) hole density distribution in N-pillar.

When the RC-IGBT with superjunction structure is functioning in bipolar mode, the variation trends of carrier concentration in pillars with different doping concentrations are the same in both Con-SJ-RC-IGBTs and PSC-SJ-RC-IGBTs. Figure 8 shows the variation trends of minority carrier concentrations in the pillars of the superjunction structure with different doping concentrations, which can effectively reflect the conductivity modulation effect. From Figure 8, in the forward conduction state, it can be observed that the minority carrier concentration rapidly decreases with increasing doping concentrations of N and P pillars. This implies that the conductivity modulation effect gradually weakens with the increasing doping concentrations of the N and P pillars. When the doping concentrations of N and P pillars reach 5 × 10^15^ cm^−3^, the minority carrier concentration becomes lower than the doping concentration. Consequently, the influence of the conductivity modulation effect on the forward voltage drop diminishes gradually, and the abrupt change phenomenon in the forward voltage drop no longer occurs in the RC-IGBT. This is also the reason why the snapback phenomenon can be mitigated in the Con-SJ-RC-IGBT with heavily doped N and P pillars. As shown in Table 2, as the doping concentrations of N and P pillars increase, the cell size required to eliminate the snapback phenomenon becomes smaller and smaller.

**Figure 9:**
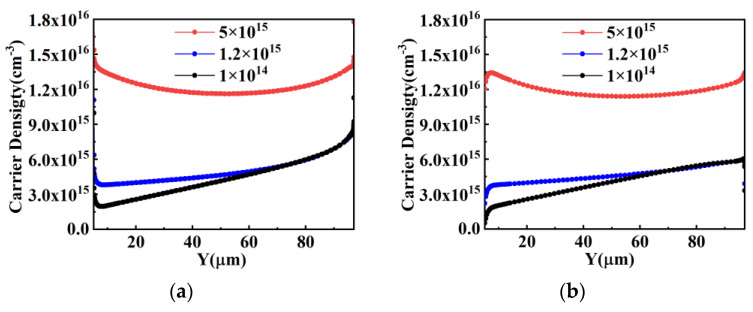
Carrier density distribution during forward conduction in (**a**) P-pillar and (**b**) N-pillar.

However, the conductivity modulation effect still exists even at high doping concentrations. The only difference is that it changes from a large injection level with low doping concentrations to a low injection level with high doping concentrations. As shown in Figure 9, the total carrier concentration during on-state in the superjunction structure still increases with the increasing doping concentrations of N and P. Therefore, the voltage drop across the device continues to decrease.

### 2.3. Reverse Conduction Characteristics

The device structure of the Con-SJ-RC-IGBT in the reverse conduction state is a superjunction PiN diode. The analysis of the device’s reverse conduction performance can also be conducted based on the carrier concentration distribution.

**Figure 10:**
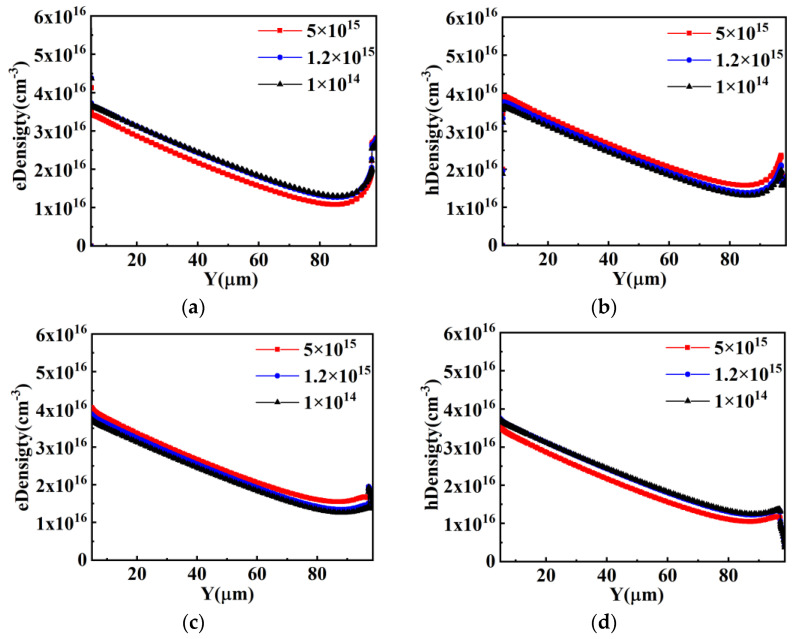
Carrier density distribution during reverse conduction: (**a**) electron density distribution in P-pillar; (**b**) hole density distribution in P-pillar; (**c**) electron density distribution in N-pillar; (**d**) hole density distribution in N-pillar.

From Figure 10, it can be observed that the impact of the conductivity modulation effect on carrier concentration enhancement is more pronounced for low doping concentrations (1 × 10^14^ cm^−3^) and moderate doping concentrations (1.2 × 10^15^ cm^−3^), where the carrier concentration is one to two orders of magnitude higher than the doping concentration. However, for high doping concentrations, the effect is minimal. As a result, the carrier concentrations under low doping concentrations approach similar levels with the carrier concentration under high doping concentrations, which means the reverse conduction voltage drop remains relatively constant regardless of variations in the doping concentrations of the P and N pillars, as shown in Figure 7c.

**Figure 11:**
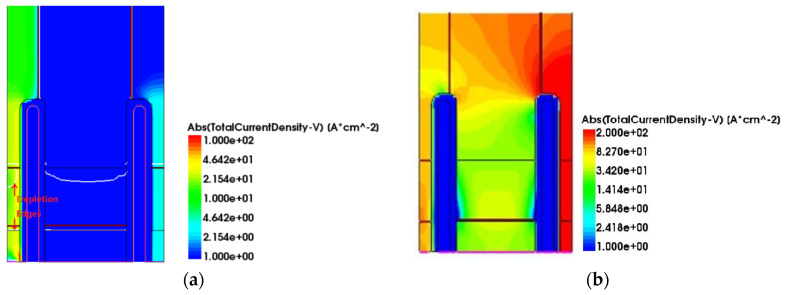
Current density distribution of PSC-SJ-RC-IGBT during reverse conduction at (**a**) J_C_ = 1 A/cm^2^ and (**b**) J_C_ = 100 A/cm^2^.

As discussed in the prior section, the reverse conduction characteristic variation trend with pillar doping concentration based on the conductivity modulation effect is similar in both two SJ-RC-IGBT devices. However, due to the presence of the P-type Schottky diode, the structure on the left side of the trench resembles a PNP structure (the p-base/N-buffer/P-Schottky) in the PSC-SJ-RC-IGBT. As shown in Figure 11, when reverse conduction occurs with a low conduction current, the P-Schottky is negatively biased, since the left diode pitch is small, the n-buffer region is depleted in both lateral and horizontal directions, and the PNP structure is triggered by punching through the n-buffer region, as shown in Figure 11a, and holes can flow through the depleted n-region to the contact, so the current is primarily composed of holes. As the current density gradually increases, the right-side PiN diode is in the on-state, the PiN diode region becomes the main current conduction path.

### 2.4. Switching Characteristics

#### 2.4.1. Reverse Recovery

**Figure 12:**
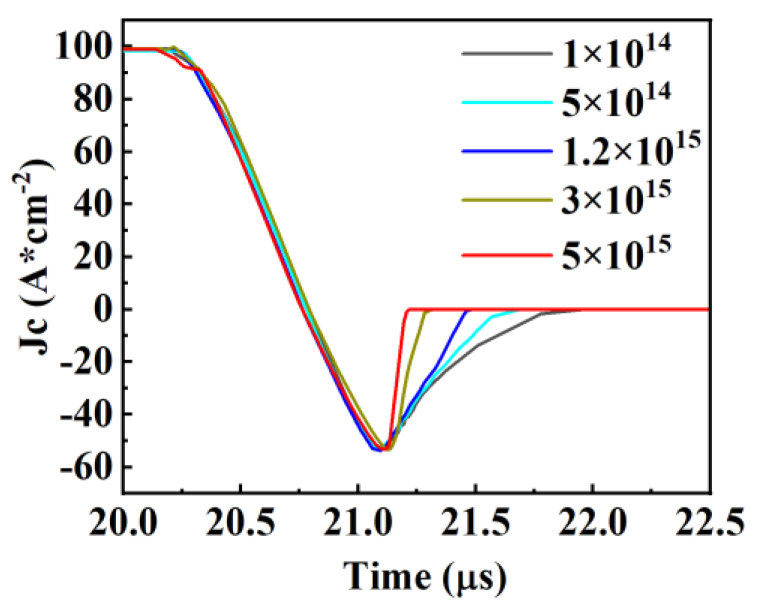
Reverse recovery characteristics of Con-SJ-RC-IGBT.

Figure 12 illustrates the trend of the Con-SJ-RC-IGBT’s reverse recovery characteristics with varying N and P pillar doping concentrations. As indicated by the previous analysis, the conductivity modulation effect weakens with increasing N and P column doping concentrations. Consequently, during reverse recovery, the excess carrier concentration in the drift region decreases with higher N and P column doping concentrations, resulting in a reduction in the total reverse recovery charge. This trend is also appropriate in PSC-SJ-RC-IGBT.

**Figure 13:**
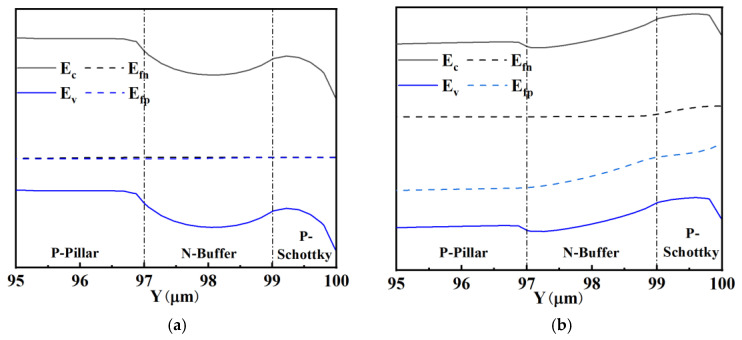
Band structure of P-type Schottky contact: (**a**) V_M_ = V_S_ and (**b**) V_M_ < V_S_.

Figure 13 is the band structure of the P-type Schottky contact at the bottom region under different biased conditions. When the applied voltage is 0, the Schottky barrier height at the bottom is high enough to prevent carriers from flowing through the contact. When the metal side voltage is lower than the Silicon side (Figure 13b), the n-buffer region is depleted, the carriers drift through the n-region to the bottom and the Schottky barrier height is lowered by the applied voltage, so the P-type Schottky diode will be in the on-state.

**Figure 14:**
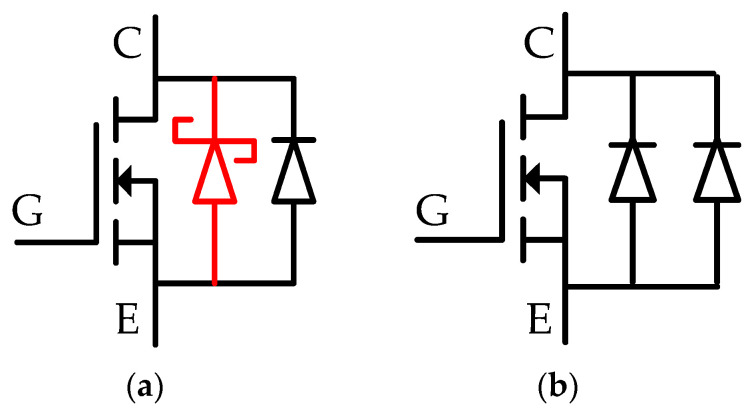
Equivalent circuit of the body diode of (**a**) PSC-SJ-RC-IGBT and (**b**) PSC-SJ-RC-IGBT without the Schottky contact.

**Figure 15:**
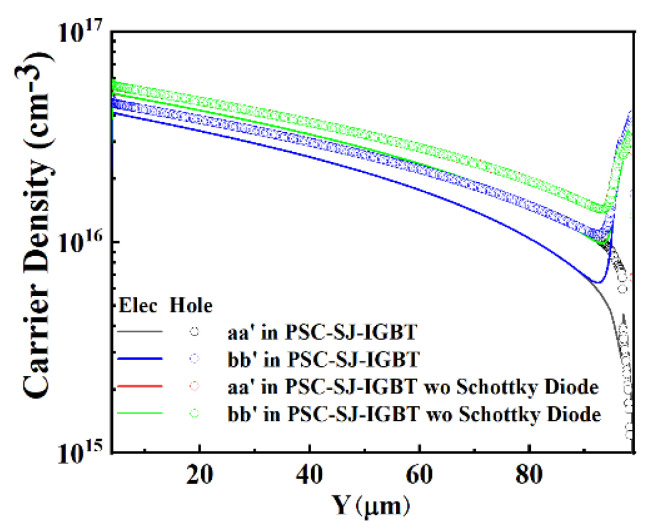
Carrier density in PSC-SJ-RC-IGBT without the Schottky diode (aa’ is the cutline along the center of left side diode; bb’ is the cutline along the center of right side diode).

The body diode in the PSC-SJ-RC-IGBT can be equivalented as a P-type Schottky diode parallel with a PiN diode, as shown in Figure 14a. Since the P-type Schottky barrier prevents electrons from injecting into the drift region of the body diode during the diode on-state, the total carrier density in the PSC-SJ-RC-IGBT is lower than the carrier density in the PSC-SJ-RC-IGBT without the Schottky contact. In Figure 15, the carriers around the Schottky contact are much lower than the Ohmic contact in PSC-SJ-RC-IGBT. The carrier density in the diode drift region of the PSC-SJ-RC-IGBT is lower than the parallel PiN diode structure (as shown in Figure 14b) in the PSC-SJ-RC-IGBT without the Schottky contact.

Since the carrier density is reduced, the voltage drop of PSC-SJ-RC-IGBT is larger than that of the Con-SJ-RC-IGBT, as shown in Figure 7c. However, as the carrier concentration in the body diode decreases, it results in a significant reduction in the reverse recovery charge. This means the reverse recovery time and reverse recovery loss is also reduced [38,39,40].

**Figure 16:**
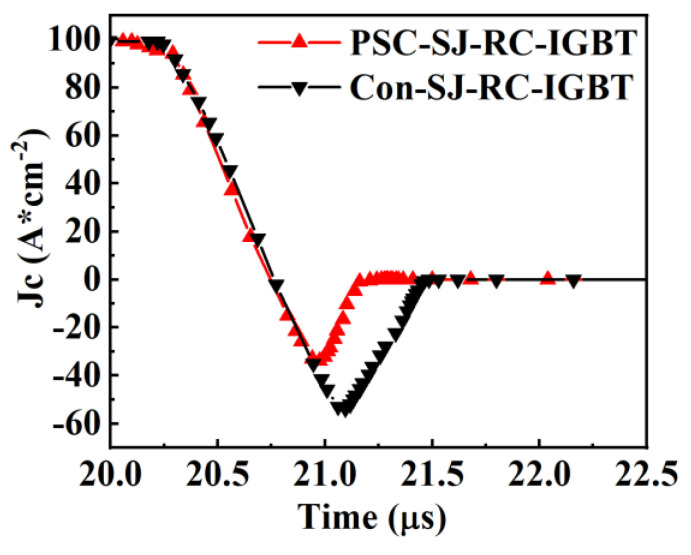
Reverse recovery performance comparison of the Con-SJ-RC-IGBT and the PSC-SJ-RC-IGBT (Pillar doping concentration = 1.2 × 10^15^ cm^−3^).

The reverse recovery characteristics of Con-SJ-RC-IGBT and PSC-SJ-RC-IGBT are presented in Figure 16. Even though the reverse recovery trends towards pillar doping concentration are similar in both two devices, the PSC-SJ-RC-IGBT exhibits a smaller reverse recovery charge (Q_rr_) due to the existence of the Schottky contact. This means that this results in a reduction in both the peak reverse recovery current (I_RRM_) and the reverse recovery time (t_rr_) compared to the Con-SJ-RC-IGBT. The recovery current (I_RRM_) for the Con-SJ-RC-IGBT is 53 A/cm^2^, while the IRRM for the PSC-SJ-RC-IGBT is 34 A/cm^2^, representing a reduction of 35%. The Con-SJ-RC-IGBT has a reverse recovery stored charge of 18.9 μC/cm^2^, whereas the PSC-SJ-RC-IGBT demonstrates a reverse recovery charge of only 8.95 μC/cm^2^, resulting in a 52.6% reduction. The reduction in stored charge also contributes to minimizing losses during the reverse recovery process.

The reverse recovery charge for the two devices under different pillar doping concentrations is listed in Table 3.

The reverse recovery charge is significantly reduced, and the Q_rr_ of PSC-SJ-RC-IGBT is almost reduced by 50% compared with Con-SJ-RC-IGBT under all pillar doping concentrations.

#### 2.4.2. E_off_ vs. V_ce_

**Figure 17:**
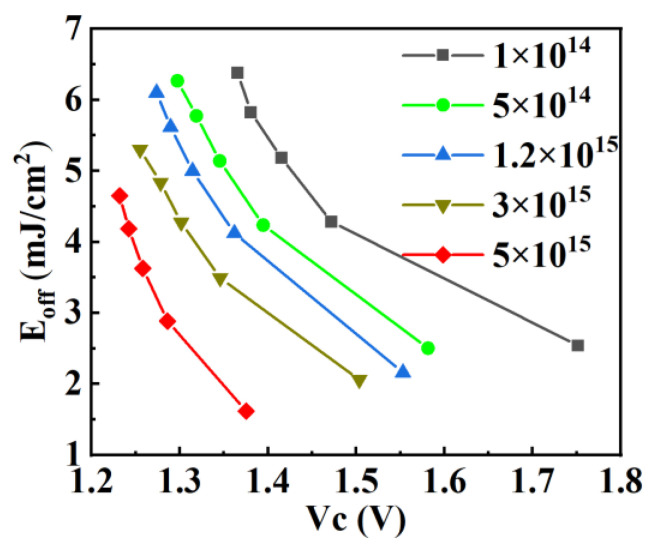
The Eoff vs. Vce trade-off in Con-SJ-RC-IGBT (J_C_ = 100 A/cm^2^).

Moreover, as the doping concentrations of N and P increase, the conductivity modulation effect weakens, resulting in a decrease in minority carrier concentration. This leads to a reduced number of carriers that need to be depleted during turn-off. Additionally, the lateral and vertical simultaneous depletion characteristic of the superjunction structure enables the quick depletion of the drift region at lower collector-emitter voltages. Therefore, the turn-off speed of the RC-IGBT with superjunction structure increases with the increasing doping concentrations of N and P, leading to lower turn-off losses. The trade-off between the conduction and turn-off losses vibration trends of the Con-SJ-RC-IGBT is shown in Figure 17; it can be observed that higher doping concentrations of N and P result in better power loss performances.

**Figure 18:**
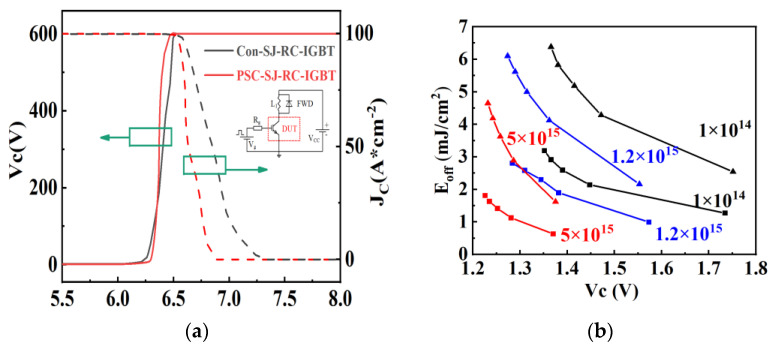
Switching-off characteristics of Con-RC-IGBT and PSC-SJ-RC-IGBT: (**a**) switching-off waves (pillar doping concentration = 1.2 × 10^15^ cm^−3^); (**b**) E_off_ vs. V_ce_ (J_C_ = 100 A/cm^2^; triangles: Con-SJ-RC-IGBT; squares: PSC-SJ-RC-IGBT).

Figure 18a illustrates the turn-off transient characteristics of the Con-SJ-RC-IGBT and PSC-SJ-RC-IGBT. It can be seen that the PSC-SJ-RC-IGBT effectively eliminates the turn-off tail current compared to the Con-SJ-RC-IGBT. The turn-off time of the Con-SJ-RC-IGBT is 0.74 μs, while the turn-off time of the PSC-SJ-RC-IGBT is only 0.38 μs, which is equivalent to 51.3% of the turn-off time of the Con-RC-IGBT. Consequently, the turn-off power consumption is significantly reduced. The turn-off loss of the Con-SJ-RC-IGBT is 4.99 mJ/cm^2^, whereas the turn-off loss of the PSC-SJ-RC-IGBT is merely 2.30 mJ/cm^2^, resulting in a reduction of 54.1%.

Even though the two devices have a similar power loss variation trends regarding pillar doping concentration (variation trends shown in Figure 14), the power loss trade-off performance of PSC-SJ-RC-IGBT is superior to that of the Con-SJ-RC-IGBT, as shown in Figure 18b.

## 3. Results

This paper investigates the advantages and issues associated with the application of the superjunction (SJ) structure in RC-IGBTs by analyzing the carrier distribution of the device during operation for the first time. The implementation of the superjunction structure in the RC-IGBT mitigates snapback more effectively as the doping concentration increases. This results in a lower reverse recovery charge and faster reverse recovery speed. The trade-off relationship between on-state voltage drop and turn-off losses is also improved. However, the snapback phenomena still exist, and the requirements for doping concentration for achieving charge balance sensitivity and minimizing power loss are completely contrary. To achieve minimum power loss, high doping concentrations are required, while optimal charge balance sensitivity necessitates low doping concentrations. Thus, a trade-off in doping concentration is required for optimal performance.

To address these issues and improve the performance of SJ-RC-IGBT, a novel Partial Schottky Collector Superjunction Reverse Conduction IGBT (PSC-SJ-RC-IGBT) is proposed, which eliminates the snapback phenomenon. By utilizing the Schottky contact and trench-shorted collector, electron extraction from the drift region is accelerated during turn-off, leading to a reduction in the turn-off loss of the device. Compared to the conventional superjunction RC-IGBT (Con-SJ-RC-IGBT), the power loss tradeoff with medium pillar doping concentration (1.2 × 10^15^ cm^−3^) is comparable to that of the Con-SJ-RC-IGBT with high pillar doping concentration (5 × 10^15^ cm^−3^). This means the charge balance sensitivity and power loss tradeoff versus pillar doping concentration is significantly improved. Additionally, the Schottky diode minimizes minority carrier injection during reverse recovery, resulting in a reduction of about 50% in reverse recovery charge compared to the Con-SJ-RC-IGBT under all pillar doping concentrations.

## Figures and Tables

**Table 1:** Key parameters of conventional RC-IGBT and SC-SJ-RC-IGBT.

Parameter	Con-RC-IGBT	SC-SJ-RC-IGBT	PSC-SJ-RC-IGBT
Cell pitch/μm	50	50	5
Thickness/μm	120	100	100
Drift region Dop/cm^−3^	1 × 10^14^	/	
N/P pillars Dop/cm^−3^	/	1 × 10^14^~5 × 10^15^	1 × 10^14^~5 × 10^15^
N/P pillars width/μm	/	2.5	2.5
FS layer thickness/μm	5	5	5
FS layer Dop/cm^−3^	1 × 10^16^	1 × 10^16^	1 × 10^16^
Collector thickness/μm	1	1	1
Collector Dop/cm^−3^	1 × 10^17^	1 × 10^17^	1 × 10^17^
Gate trench width/μm	1	1	1
N collector length/μm	5	5	1

**Table 2:** Cell pitch to eliminate snapback vs. pillar doping concentration.

Pillar Doping Concentration (cm^3^)	Cell Pitch to Eliminate Snapback (μm)
1 × 10^14^	600
5 × 10^14^	200
1.2 × 10^15^	100
3 × 10^15^	50
5 × 10^15^	20

**Table 3:** Reverse recovery charge vs. pillar doping concentration.

Pillar Doping Concentration (cm^3^)	Con-SJ-RC-IGBT Qrr (μC/cm^2^)	PSC-SJ-RC-IGBT Qrr (μC/cm^2^)
1 × 10^14^	24.46	12.05
5 × 10^14^	22.30	11.37
1.2 × 10^15^	18.90	8.95
3 × 10^15^	14.46	7.95
5 × 10^15^	13.45	7.67

## Data Availability

Data are contained within the article.
